# Effects of SNPs (CYP1B1*2 G355T, CYP1B1*3 C4326G, and CYP2E1*5 G-1293C), Smoking, and Drinking on Susceptibility to Laryngeal Cancer among Han Chinese

**DOI:** 10.1371/journal.pone.0106580

**Published:** 2014-10-09

**Authors:** Jianhua Jin, Faming Lin, Shiyu Liao, Qiyu Bao, Liyan Ni

**Affiliations:** 1 School of Basic Medicine, Wenzhou Medical University, Wenzhou, China; 2 The Second Affiliated Hospital of Wenzhou medical University, Wenzhou, China; 3 Institute of Biomedical Informatics/Zhejiang Provincial Key Laboratory of Medical Genetics, School of Life Sciences, Wenzhou Medical University, Wenzhou, China; Duke Cancer Institute, United States of America

## Abstract

**Purpose:**

This study was conducted to explore the effects of genetic polymorphisms (CYP1B1*2 G355T, CYP1B1*3 C4326G, and CYP2E1*5 G-1293C) and environmental factors (smoking and drinking) on susceptibility to laryngeal cancer in a Han Chinese study group.

**Methods:**

This case-control study included 552 Han Chinese patients diagnosed with laryngeal cancer and 666 healthy control subjects of the same ethnicity, similar age, and gender. Genetic polymorphisms were examined using multi-PCR and Matrix Assisted Laser Desorption Ionization - Time of Flight (MALDI-TOF MS) methodology. The association of these genetic and environmental factors with susceptibility to laryngeal cancer was evaluated using a statistical approach.

**Results:**

The frequencies of all three polymorphisms in the patient cohort were significantly different from those in the control cohort. Compared to the control cohort, carriers of variant alleles of CYP1B1*2 355T and CYP2E1*5 -1293C showed a higher risk for developing laryngeal cancer (for CYP1B1*2 355T, adjusted OR = 2.657, P <0.001; for CYP2E1*5 -1293C, adjusted OR = 1.938, P <0.001), while carriers of mutation allele CYP1B1*3 4326G showed a lower risk (adjusted OR = 0.562, P <0.001). Joint effects of these polymorphisms were observed. When compared to haplotype G_355_C_4326_G_−1293_, haplotypes T_355_C_4326_G_−1293_ (adjusted OR = 1.809, P <0.001), G_355_C_4326_C_−1293_ (adjusted OR = 1.644, P = 0.044), and T_355_C_4326_C_−1293_ (adjusted OR = 3.104, P <0.001) were associated with a significantly higher laryngeal cancer risk. The adjusted ORs for non-smokers, non-drinkers, smokers, and drinkers with the GT/TT genotype at CYP1B1*2 G355T were 2.190 (P = 0.006), 2.008 (P = 0.001), 5.875 (P <0.001), and 4.518 (P <0.001), respectively.

**Conclusions:**

CYP1B1*2 355T and CYP2E1*5 -1293C are associated with an increased laryngeal cancer risk, while CYP1B1*3 4326G is associated with a decreased risk. These polymorphisms showed joint effects on laryngeal cancer risk. Smoking and drinking showed collaborative effects with two high risk alleles (CYP1B1*2 355T and CYP1B1*3 4326G) for promoting laryngeal cancer risk.

## Introduction

Laryngeal cancer, the most common malignant head neck cancer, is also a common malignant tumor in China [1]; however, the etiology of laryngeal cancer remains unclear. While the majority of laryngeal cancer patients have a long history of smoking and alcohol assumption [2], only a small percentage of such individuals eventually develop laryngeal cancer. This suggests that an individual's genetic make-up plays an important role their susceptibility to laryngeal cancer [3], and supports the current notion that susceptibility to laryngeal cancer is associated with interactions between genes and the environment. Therefore, use of molecular genetic approaches and genetic association analysis to identify genes potentially related to laryngeal cancer should assist in revealing the etiology of the disease.

CYP1B1 and CYP2E1 are two cytochrome P450 enzymes which catalyze the hydroxylation of pro-carcinogens such as polycyclic aromatic hydrocarbons (PAHs), heterocyclic aromatic amines (HAAs), aromatic amines, and N-nitrosamines [4]–[7]. These reactions may produce cytotoxic agents, induce mutations in proto-oncogenes or tumor suppressor genes, result in DNA damage, intensify lipid and protein peroxidation, and ultimately lead to an increased risk for developing various cancers [8], [9]. Currently, > 50 polymorphic variants have been reported to affect encoding of the CYP1B1 protein, and among these, four single nucleotide polymorphisms (SNPs) (CYP1B1*2 C142G, CYP1B1*2 G355T, CYP1B1*3 C4326G, and CYP1B1*4 A4390G) result in changes in the amino composition of the enzyme and thereby affect metabolism of carcinogens [10], [11]. The SNP CYP1B1*3 (Leu^432^) has been reported to result in a higher catalytic activity for oxidation of benzo[α]pyrene to 7,8-dihydoxy-7,8-dihydrodiol, compared to the Val^432^ form of the enzyme [12]. Helmig et al. conducted a study investigating the association between gene-environment interactions and cancer susceptibility, and reported that smokers had a high frequency of wild-type allele C at the nucleotide 4326 (nt4326) locus of CYP1B1 [13]. Studies of human head and neck squamous-cell cancer (HNSCC) revealed that polymorphism CYP1B1*3 C4326G is associated with tobacco exposure [14]. Singh et al. reported that while CYP1B1*2 G355T and CYP1B1*3 C4326G both increased an individual's susceptibility to HNSCC, susceptibility was also affected by interactions between genes and environmental factors (mainly tobacco and alcohol exposure) [15]. Furthermore, an association between these SPNs and other squamous-cell cancers has also been suggested. A meta-analysis conducted by Xu et al. found that homozygous mutations of CYP1B1*2 G355T and CYP1B1*3 C4326G may be low penetrance risk factors for lung cancer occurrence [16], which is consistent with results reported by Chen et al. [17]. Additionally, CYP1B1*2 G355T and CYP1B1*3 C4326G have also been reported to be associated with susceptibility to hormone-related cancers such as prostate cancer, bladder cancer, and carcinoma of the endometrium [18]–[20].

While a G-1293C mutation at the 5′ promoter region of CYP2E1 will alter the gene's expression [21], the impact of this SNP on an individual's risk for developing head and neck cancer remains controversial. Gajeka et al. reported that CYP2E1 was not associated with susceptibility to either laryngeal cancer or nasopharyngeal carcinoma [22], and Cury et al. reported that CYP2E1 was not associated with susceptibility to head and neck cancer [23]. However, it was reported that the presence of mutation allele CYP2E1*5 may increase the risk for developing HNSCC, and a joint effect between this genetic polymorphism and environment factors such as smoking and alcohol consumption was also noticed [24].

Here, we report results of a case-control study investigating the association of three important SNPs of P450 (CYP1B1*2 G355T, CYP1B1*3 C4326G, and CYP2E1*5 G-1293C) and two major environmental factors (smoking and drinking) with susceptibility to laryngeal cancer. The goal of this study was to improve help improve the early diagnosis, and ultimately reduce the incidence of laryngeal cancer in a high risk population in China.

## Study Subjects and Methods

### Study subjects

The experimental protocol was established, according to the ethical guidelines of the Helsinki Declaration. This study was approved by the Institutional Review Board of the Second Affiliated Hospital and Yuying Children's Hospital of Wenzhou Medical University, and all subjects in both cohorts signed an Informed Consent prior to enrollment. The patient cohort in this study included 552 Han Chinese individuals diagnosed with laryngeal cancer between August 2007 and August 2014 at various hospitals affiliated with Wenzhou Medical College (WMC). Subjects were eligible for inclusion in the study based on the following criteria: (1) a diagnosis of laryngeal squamous-cell cancer as a primary disease based on a histopathology examination; (2) the availability of a peripheral blood specimen that was collected prior to radiochemotherapy and was properly preserved; (3) the availability of complete clinical records; (4) the subject was not related to other Han Chinese patients. The control cohort included 666 healthy individuals who had undergone a routine health checkup during the same time period at the Second Hospital affiliated with WMC. The selection criteria used for the control cohort were the same as those used for the patient cohort, except that the control subjects were required not to have a personal history of cancer, a family history of cancer, or a history of exposure to radioactive or toxic gas or other known carcinogens. The patient and control cohorts were matched for gender and age, and there was no statistically significant difference between their demographic characteristics. This study was approved by the Institutional Review Board of the Second Hospital affiliated with WMC, and all subjects in both cohorts signed an Informed Consent prior to enrollment.

### Primer design

Primers for the Multiplex Polymerase Chain Reaction (Multi-PCR) were designed by the Assay Design 3.1 system (Sequenom, Inc., San Diego, CA, USA) and synthesized by TsingKe Inc, (Beijing, China). The primer sequences used in this study are listed in Table 1.

**Table 1:** PCR primer sequences for SNP amplification and extension.

dbSNP ID	HGVS nomenclature	SNP	PCR primer	
rs1056827	CYP1B1*2	G355T	F	ACGTTGGATGGACACCACACGGAAGGAGG
			R	ACGTTGGATGTAGTGGTGCTGAATGGCGAG
			E	AACACACGGAAGGAGGCGAAGG
rs1056836	CYP1B1*3	C4326G	F	ACGTTGGATGCTTGTCCAAGAATCGAGCTG
			R	ACGTTGGATGCAACCAGTGGTCTGTGAATC
			E	CTCTCCGGGTTAGGCCACTTCA
rs3013867	CYP2E1*5	G1293C	F	ACGTTGGATGAAACCAGAGGGAAGCAAAGG
			R	ACGTTGGATGTTGGTTGTGCTGCACCTAAC
			E	TTCTTGGTTCAGGAGAG

F  =  Forward; R  =  Reverse; E  =  Extension.

### Genomic DNA extraction

Specimens of peripheral blood were collected in EDTA-coated tubes and stored at −70°C. DNA was extracted from blood cells using the AxyPrep Blood Genomic DNA Maxiprep Kit (Axygen BioScience, Inc., Union City, CA, USA).

### Gene amplification and polymorphism analysis:

DNA regions containing targeted SNPs were amplified using Multi-PCR with the GeneAMP PCR System 9700 (Applied Biosystems, Inc., Carlsbad, CA, USA). Conditions for PCR were as follows: initial incubation at 95°C for 2 min, followed by 45 cycles of 95°C for 30 s and annealing at 56°C for 30 s and 72°C for 60 s. Final extension was conducted by incubation at 72°C for 5 min. The PCR products were purified by addition of SAP enzyme, which was used as a template for amplification of SNPs by primer extension reactions with ddNTP. The primer extension was performed using the following sequential steps: initial incubation at 95°C for 30 s; denaturation at 95°C for 5 s; five cycles of annealing at 52°C for 5 s and extension at 80°C for 5 s; 40 cycles of denaturation at 95°C for 5 s, annealing at 52°C for 5 s and extension at 80°C for 5 s; final extension at 72°C for 53 min. Genotyping of extension reaction products was done using matrix assisted laser desorption ionization time of flight MS (MLDI-TOF MS) [25].

### Statistical analysis

The Hardy–Weinberg equilibrium was used to determine whether the genotype and allele frequencies in both the patient and the control cohorts were representative of frequencies in the overall population. Evaluations of haplotype construction and linkage disequilibrium were conducted using Haploview 3.2 software (http://www.broad.mit.edu/mpg/haploview/). Differences in genotype and allele frequencies were examined using the chi squared (χ^2^) test. Adjusted ORs based on age, gender, smoking habits, and drinking status were calculated using logistic regression and unconditional logistic regression analyses. Unconditional logistic regression analysis was used to access the combined effects of SNPs and tobacco/alcohol exposure [26], [27].

Smoking was quantified using the smoking index formula (SI  =  pack-year  =  number of cigarettes per day/20 x years of smoking), and individuals were classified as light or heavy smokers based on SI values of ≤ 20 or > 20, respectively [28]. Drinking was quantified using the drinking index (DI  =  mL of drinks per day x years of drinking), and individuals were classified as light or heavy drinkers based on DI values of ≤ 60 or > 60, respectively [29].

All statistical analyses were performed using SPSS 20.0 software. A P-value <0.05 was considered statistically significant.

## Results

### Subject characteristics

Demographic information for the study subjects in both groups is summarized in Table 2. There were no statistically significant differences regarding the gender (P = 0.643) or age (mean = 63.5 years in patient group vs. 62.3 years in control group, P = 0.678) of the subjects in the two groups. However, males accounted for 96.7% of the subjects enrolled in the patient cohort (P <0.001). The majority of subjects in both groups reported a long history of smoking and/or drinking, but there were more smokers in the patient cohort than in the control cohort (86.4% vs. 65.6%, P <0.001). Heavy smokers showed a significantly (P <0.001) increased risk for laryngeal cancer (OR = 5.552, 95% CI: 4.069–7.574), while the risk for light smokers was not significant (OR = 1.299, 95% CI: 0.913–1.848, P = 0.164). The effect of drinking alcohol on the risk for laryngeal cancer was similar to that produced by smoking (P <0.001), and there were more drinkers in the patient cohort than in the control cohort (69.4% vs. 49.4%, respectively, P <0.001). Additionally, heavy drinking was associated with a significant risk for developing laryngeal cancer (OR = 4.085, 95% CI: 3.113–5.361, P <0.001), whereas this risk among light drinkers was not significant (OR = 0.949, 95% CI: 0.693–1.298, P = 0.741).

**Table 2:** Characteristics of patients and control subjects.

Variable	Patients, n. (%)	Controls, n. (%)		
Sample size	N = 552	N = 666	Crude OR (95% CI)	*P*
Mean age	63.5 (10.4)	62.3 (9.4)		0.678
≤ 50	53 (9.6)	27 (4.1)		
> 50	499 (90.4)	639 (95.9)		
Gender				0.643
Male	534 (96.7)	641 (96.2)		
Female	18 (3.3)	25 (3.8)		
Smoking status				<0.001
Nonsmoker	75 (13.6)	229 (34.4)	1(Ref.)	
Smoker	477 (86.4)	437 (65.6)		
≤ 20 pk-yr	97 (17.6)	228 (34.2)	1.299 (0.913–1.848)	0.146
> 20 pk-yr	380 (68.8)	209 (31.4)	5.552 (4.069–7.574)	<0.001
Drinking status				<0.001
No	169 (30.6)	337 (50.6)	1 (Ref.)	
Yes	383 (69.4)	222 (49.4)		
≤ 60 DI	88 (15.9)	185 (27.8)	0.949 (0.693–1.298)	0.741
> 60 DI	295 (53.4)	144 (21.6)	4.085 (3.113–5.361)	<0.001
Laryngeal cancer stage				
I	108 (19.6)			
II	142 (25.7)			
III	156 (28.3)			
IV	146 (26.4)			

Ref: used as reference values. OR: Odds Ratio; 95% CI: 95% confidence interval; Stage of laryngeal cancer was classified using the UICC-2002 TNM classification system.

### Allele and genotype distribution

Allele and genotype frequencies for the three SNP loci (CYP1B1*2 G355T, CYP1B1*3 C4326G, and CYP2E1*5 G-1293C) are summarized in Table 3. The distributions of the allele and genotype frequencies of these three loci were consistent with the Hardy-Weinberg equilibrium (χ^2^ = 0.270, P = 0.603; χ^2^ = 0.045, P = 0.832; χ^2^ = 3.127, P = 0.077; respectively). The allele frequencies for CYP1B1*2 355T, CYP1B1*3 4326G, and CYP2E1*5 -1293C were 35.2, 8.2, and 15.5%, respectively in the patient cohort, and 20.2, 13.2, and 8.3%, respectively in the control cohort (P <0.05). The frequencies of the genotypes at these three loci in the two cohorts were significantly different (P <0.001, respectively), and the frequencies of the wild-type genotype in the patient and control cohorts were also significantly different (64.5% vs. 36.6%, P <0.001; 15.2% vs. 24.8%, P <0.001; 20.3% vs. 15.3%, P <0.001, respectively).

**Table 3:** Distribution of SNPs in patient and control cohorts.

		Patients, n. (%)	Controls, n. (%)	OR^c^ (95% CI)	P	OR^a^ (95% CI)	P^a^
rs1056827	G>T Genotype	552	666		<0.001		<0.001
	GG	196 (35.5)	422 (63.4)	1(Ref.)			
	GT	322 (58.3)	219 (32.9)	3.166 (2.487–4.029)	<0.001	2.721 (2.114–3.501)	<0.001
	TT	34(6.2)	25(3.8)	2.928 (1.700–5.042)	<0.001	2.134 (1.221–3.073)	0.008
	GT+TT	356 (64.5)	244 (36.6)	3.141 (2.483–3.974)	<0.001	2.657(2.078–3.398)	<0.001
	Allele						<0.001
	G	714 (64.8)	1063 (79.8)				
	T	390 (35.2)	269 (20.2)				
rs1056836	C>G Genotype	552	666		<0.001		<0.001
	CC	468 (84.8)	501 (75.2)	1 (Ref.)			
	CG	77 (13.9)	154 (23.1)	0.535 (0.396–0.723)	0.001	0.532 (0.388–0.729)	<0.001
	GG	7 (1.3)	11 (1.7)	0.681 (0.262–1.772)	0.431	1.195 (0.433–3.299)	0.731
	CG+GG	85 (15.2)	165 (24.8)	0.545 (0.407–0.729)	<0.001	0.562 (0.414–0.763)	<0.001
	Allele						<0.001
	C	1013 (91.8)	1156 (86.8)				
	G	91 (8.2)	176 (13.2)				
rs3813867	G>C Genotype	552	666		<0.001		<0.001
	GG	418 (75.7)	564 (84.7)	1 (Ref.)			
	GC	97 (17.6)	94 (14.1)	1.392 (1.021–1.889)	0.037	1.485 (1.069–2.064)	0.018
	CC	37 (6.7)	8 (1.2)	6.240 (2.876–13.540)	<0.001	8.718(3.785–20.076)	<0.001
	GC+CC	134 (20.3)	102 (15.3)	1.773 (1.330–2.362)	<0.001	1.938 (1.426–2.633)	<0.001
	Allele						<0.001
	G	933 (84.5)	1222 (91.7)				
	C	171 (15.5)	43 (8.3)				

Ref: used as reference values. OR^C^: Crude Odds Ratio; OR^a^: Odds Ratio adjusted by logic regression based on age, gender, smoking, and drinking status; 95% CI: 95% confidence interval.

### Individual SNPs and risk of laryngeal cancer

Individuals with the GT or TT (GT+TT) genotype at the CYP1B1*2 G355T locus showed a significantly higher risk for developing laryngeal cancer than individuals with the GG genotype (crude OR = 3.141, 95% CI: 2.483–3.974, P <0.001), and this difference between genotype groups remained significant even after adjusting for differences in age, gender, smoking habits, and drinking habits (adjusted OR = 2.657, 95% CI: 2.078–3.398, P <0.001).

In contrast, individuals with the CG or GG genotype at the CYP1B1*3 C4326G locus showed a significantly lower risk for developing laryngeal cancer compared to individuals with the CC genotype (crude OR = 0.545, 95% CI: 0.407–0.729, P <0.001). Adjusted values also showed statistical significance: (adjusted OR = 0.562, 95% CI: 0.414–0.763, P <0.001).

Individuals with the GC or CC genotype at the CYP2E1*5 G-1293C locus also showed a significantly higher risk of laryngeal cancer compared to individuals with genotype GG (crude OR = 1.773, 95% CI: 1.330–2.362, P <0.001), while individuals heterozygous for genotype CC showed a much higher risk (crude OR = 6.240, 95% CI: 2.876–13.540, P <0.001). The adjusted values also showed statistical significance: (for GC + CC, adjusted OR = 1.938, 95% CI: 1.426–2.633, P <0.001; for CC, adjusted OR = 8.718, 95% CI: 3.785–20.076, P <0.001).

### Joint effect of SNPs on susceptibility to laryngeal cancer

The distributions of haplotypes in the two cohorts and results of relevant statistical analyses are summarized in Table 4. Six of the eight haplotypes possibly associated with laryngeal cancer were identified in the patient and control groups. The most common haplotype, G_335_C_4326_G_−1293_, was found in 56.4% of patients and 67.7% of control subjects. Six haplotypes were found to be associated with a susceptibility to laryngeal cancer. For example, the T_355_C_4326_G_−1293_ haplotype, which contains two high-risk alleles (T355 and C4326), showed a stronger association with laryngeal cancer than the major haplotype G_335_C_4326_G_−1293_ (adjusted OR = 1.809, 95% CI: 1.434–2.282, P <0.001). Additionally, the T_355_C_4326_C_−1293_ haplotype, which contains three high risk alleles, showed an even stronger association (adjusted OR = 3.104, 95% CI: 2.135–4.512, P <0.001). Accordingly, the haplotype G_355_G_4326_G_−1293_, which contains three protective alleles, showed a weaker association with laryngeal cancer than the major haplotype G_335_C_4326_G_−1293_ (adjusted OR = 0.573, 95% CI: 0.391–0.841, P = 0.004).

**Table 4:** Haplotype and genotype distributions in patient and control cohorts.

Chromosomes	Patients, n.%	Controls, n.%	OR^a^ (95% CI)	P^a^
Haplotypes	1104	1332		
G_355_C_4326_G_−1293_	623 (56.4)	902 (67.7)	1 (Ref.)	
G_355_G_4326_G_−1293_	42 (3.8)	111 (8.3)	0.573 (0.391–0.841)	0.004
G_355_G_4326_C_−1293_	9 (0.8)	8 (0.6)	4.264(1.569–11.592)	0.004
T_355_C_4326_G_−1293_	243 (22.0)	165 (12.4)	1.809 (1.434–2.282)	<0.001
G_355_C_4326_C_−1293_	40 (3.6)	42 (3.1)	1.644 (1.014–2.666)	0.044
T_355_C_4326_C_−1293_	107 (9.7)	47 (3.5)	3.104 (2.135–4.512)	<0.001
genotypes	552	666		
GG+CG/GG+GG	39 (7.1)	100 (15.0)	1 (Ref.)	
GT/TT+CC+GG	216 (39.1)	142 (21.3)	3.331 (2.144–5.176)	<0.001
GT/TT+CG/GG + GC/CC	15 (2.7)	13 (1.9)	2.418 (0.999–5.856)	0.050
GG+CG/GG+ GC/CC	5 (0.9)	8 (1.2)	3.323(0.964–11.450)	0.057
GT/TT+CC+GC/CC	100 (18.1)	45 (6.8)	5.297(3.123–8.986)	<0.001

Ref: used as reference values. OR^C^: Crude Odds Ratio; OR^a^: Odds Ratio adjusted by logic regression based on age, gender, smoking, and drinking status; 95% CI: 95% confidence interval.

Genotypes at the three loci were defined for individuals in the patient and control cohorts. Individuals with genotype GT/TT+CC+GG or GT/TT+CG/GG + GC/CC, which contains two risk alleles at two loci, showed a significantly higher risk for developing laryngeal cancer compared to individuals with genotype GG+CG/GG+GG (adjusted OR = 3.331, 95% CI: 2.144–5.176, P <0.001 and adjusted OR = 2.418, 95% CI: 0.999–5.856, P = 0.050, respectively). Moreover, individuals with three risk alleles at all three loci (GT/TT+CC+GC/CC) showed the highest risk for developing laryngeal cancer (adjusted OR = 5.297, 95% CI: 3.123–8.986, P <0.001).

### Gene-environment interactions and the risk of laryngeal cancer

The joint effects of the three SNPs and environmental factors are presented in Table 5. Our analysis showed that 58.5% of the patients exposed to tobacco smoking had the GT or TT genotype at the CYP1B1*2 G355T locus, and 38.3% had the genotype GG at that locus. Among patients who consumed alcohol, 47.6% had the GT or TT genotype and 21.7% had the GG genotype. Non-smokers with the GT or TT genotype showed a higher risk for developing laryngeal cancer than non-smokers with the GG genotype (adjusted OR = 2.190, 95% CI: 1.257–3.816, P = 006). The corresponding values for non-drinkers were OR = 2.008, 95% CI: 1.361–2.963, and P = 0.001, and for smokers, the values were OR = 5.875, 95% CI: 3.950–8.739, and P <0.001. This OR value, which reflects the joint effect of having the GT or TT genotype and also being a smoker, is larger than the value produced by multiplying the OR value for having the genotype alone with the OR value for being a smoker alone (5.875 > 2.190×2.098  =  4.595). Among drinkers, the corresponding values were OR = 4.518, 95% CI: 3.181–6.415, P <0.001. This OR value was also larger than the value produced by multiplying the OR value for having the genotype alone with the OR value for being a smoker alone (4.518 > 2.008×1.414  =  2.839). The OR values for heavy smokers and heavy drinkers with the GT+TT genotype were 9.995 (P <0.001) and 8.668 (P <0.001), respectively. Similar joint effects of heavy smoking or heavy drinking coupled with genetic factors were also indicated (9.995 > 2.190×3.515  =  7.700 and 8.668 > 2.008×2.312  =  4.642, respectively). These data demonstrate that the presence of an SNP at the CYP1B1*2 G355T locus, when coupled with smoking or drinking, produces a joint effect on an individual's susceptibility to laryngeal cancer. Also, the presence of the allele 335T, when coupled with smoking or drinking, collaboratively increases the risk for laryngeal cancer. Our results show that the joint effect of two risk factors is stronger than the simple additive effect of two single factors.

**Table 5:** Effect of gene-environment interaction on laryngeal cancer risk.

	G>T rs1056827					
	GG			GT+TT		
	Pat/Con, n.%	OR^a^ (95% CI)	P^a^	Pat/Con, n.%	OR^a^ (95% CI)	P^a^
Smoking status						
Nonsmoker	42 (7.6)/167 (25.1)	1(Ref.)		33 (6.0)/62 (9.3)	2.190 (1.257–3.816)	0.006
Smoker	154 (27.9)/255 (38.3)	2.098 (1.402–3.140)	<0.001	323 (58.5)/182 (27.3)	5.875 (3.950–8.739)	<0.001
≤ 20 pk-yr	43 (7.8)/139 (20.9)	0.990 (0.601–1.629)	0.968	54 (9.8)/89 (13.4)	1.736 (1.048–2.875)	0.032
> 20 pk-yr	111 (20.1)/116 (17.4)	3.515 (2.265–5.457)	<0.001	269 (48.7)/93 (14.0)	9.995 (6.514–15.336)	<0.001
Alcohol drinking						
No	76 (13.8)/221 (33.2)	1(Ref.)		93 (16.8)/116 (17.4)	2.008(1.361–2.963)	0.001
Yes	120 (21.7)/72 (30.2)	1.414 (0.989–2.020)	0.057	263 (47.6)/76 (19.2)	4.518 (3.181–6.415)	<0.001
≤ 60 DI	27 (4.9)/109 (16.4)	0.616 (0.371–1.021)	0.060	61 (11.1)/76 (11.4)	1.752(1.126–2.725)	0.013
> 60 DI	93 (16.8)/92 (13.8)	2.312 (1.548–3.454)	<0.001	202 (36.6)/52 (7.8)	8.668 (5.728–13.116)	<0.001

	C>G rs1056836					
	CC			CG+GG		
	Pat/Con, n.%	OR^a^ (95% CI)	P^a^	Pat/Con, n.%	OR^a^ (95% CI)	P^a^
Smoking status						
Nonsmoker	60 (10.9)/173 (26.0)	1(Ref.)		15 (2.3)/56 (8.4)	0.735 (0.380–1.422)	0.360
Smoker	408 (73.9)/328 (49.2)	2.959 (2.107–4.154)	<0.001	69 (10.4)/109 (16.4)	1.542 (1.001–2.376)	0.050
≤ 20 pk-yr	85 (15.4)/176 (26.4)	1.050 (0.696–1.584)	0.816	12 (1.8)/52 (7.8)	0.408 (0.190–0.873)	0.021
> 20 pk-yr	323 (58.5)/152 (22.8)	5.183 (3.598–7.467)	<0.001	57 (8.6)/57 (8.6)	2.685 (1.659–4.344)	<0.001
Alcohol drinking						
No	132 (23.9)/250 (37.5)	1(Ref.)		37 (6.7)/87 (13.1)	0.749 (0.474–1.183)	0.215
Yes	336 (60.9)/251 (37.7)	2.047 (1.547–2.710)	<0.001	47 (8.5)/78 (11.7)	0.930 (0.604–1.432)	0.930
≤ 60 DI	70 (12.7)/148 (22.2)	0.750 (0.520–1.083)	0.125	18 (3.3)/37 (5.6)	0.751 (0.406–1.139)	0.362
> 60 DI	266 (48.2)/103 (15.5)	3.897 (2.821–5.382)	<0.001	29 (5.3)/41 (6.1)	1.111 (0.652–1.895)	0.698

	G>C rs3813867					
	GG			GC+CC		
	Pat/Con, n.%	OR^a^ (95% CI)	P^a^	Pat/Con, n.%	OR^a^ (95% CI)	P^a^
Smoking status						
Nonsmoker	52 (9.4)/188 (28.2)	1 (Ref.)		23 (4.2)/41 (6.2)	2.509 (1.363–4.621)	0.003
Smoker	366 (66.3)/376 (56.5)	3.044 (2.143–4.322)	<0.001	111 (20.1)/61 (9.2)	5.508 (3.508–8.648)	<0.001
≤ 20 pk-yr	65 (11.8)/205 (30.8)	0.921 (0.312–8.719)	0.711	32 (5.8)/23 (3.5)	3.409 (1.776–6.543)	<0.001
> 20 pk-yr	301 (54.5)/171 (25.7)	5.764 (3.960–8.391)	<0.001	79 (14.3)/38 (5.7)	7.029 (4.224–11.694)	<0.001
Alcohol drinking						
No	115 (20.8)/291 (43.7)	1(Ref.)		54 (9.8)/46 (6.9)	3.630(2.254–5.847)	<0.001
Yes	303 (54.9)/273 (41.0)	2.136 (1.515–3.012)	<0.001	80 (14.5)/56 (5.6)	2.099 (1.094–4.028)	0.026
≤ 60 DI	65 (11.8)/145 (21.8)	0.990 (0.680–1.440)	0.956	23 (4.2)/40 (6.0)	1.162 (0.654–2.066)	0.608
> 60 DI	238 (43.1)/128 (19.2)	3.891 (2.840–5.331)	<0.001	57 (10.3)/16 (2.4)	7.414 (4.040–13.604)	<0.001

Ref: used as reference values. OR^C^: Crude Odds Ratio; OR^a^: Odds Ratio adjusted by logic regression based on age, gender, smoking, and drinking status; 95% CI: 95% confidence interval.

Similarly, both smoking and drinking showed a collaborative effect for increasing the risk of laryngeal cancer in patients with the wild type allele 4326C at the CYP1B1*3 C4326G locus. For instance, individuals with the CG+GG genotype at this locus presented with a lower risk for laryngeal cancer than the those with the CC genotype and experiencing the same environmental exposures, such as smoking (OR = 1.542 vs. OR = 2.959, P <0.05). We did not observe the same collaborative effect between the SNP at the CYP2E1*5 G-1293C locus and smoking or drinking.

The results of multivariate prognostic analyses for the effects of risk factor interactions on laryngeal cancer risk are shown in Table 6. Environmental factors (smoking and drinking) and genetic factors (CYP1B1*2 355T and CYP2E1*5 -1293C) are both associated with an individual's susceptibility to developing laryngeal cancer (P value for all these evaluations were < 0.001). The mutant allele CYP1B1*2 4326G shows a protective effect against developing laryngeal cancer (P <0.001). The genetic risk factors and smoking/drinking show joint effects on laryngeal cancer occurrence.

**Table 6:** Multivariate prognostic analyses for the effect of risk factor interaction on laryngeal cancer risk.

	OR	95% CI	P
smoking	2.415	1.769–3.297	<0.001
drinking	1.765	1.366–2.279	<0.001
rs1056827	2.420	1.881–3.113	<0.001
rs1056836	0.642	0.471–0.874	0.005
rs3813867	1.465	1.066–2.013	0.019
smoke * rs1056827	2.352	1.705–3.245	<0.001
drink * rs1056827	2.081	1.479–2.929	<0.001
smoke * rs1056836	2.187	1.658–2.884	<0.001
drink * rs1056836	1.782	1.367–2.324	<0.001

The mutant genotype (CG/GG) was used as a control to calculate the interactive effect of rs1056836 and smoke/drink.

## Discussion

Our case-control study of the Han Chinese population revealed that three SNPs at CYP1B1*2 G355T, CYP1B1*2 C4326G, and CYP2E1*5 G-1293C are associated with an individual's susceptibility to laryngeal cancer. In the patient cohort, the frequencies of alleles CYP1B1*2 355T and CYP2E1*5 -1293C were higher than those in the control cohort, and the frequency of allele CYP1B1*3 4326G in the patient cohort was lower than that in the control cohort. Additionally, all of these differences were statistically significant. These results strongly suggest that alleles CYP1B1*2 355T and CYP2E1*5 -1293C increase the risk of laryngeal cancer, while allele CYP1B1*3 4326G may reduce the risk.

CYP1B1*2 G355T locates at the second exon of the CYP1B1 gene and causes an Ala119Ser amino acid change in the encoded enzyme. The CYP1B1*2 C355T mutation has been reported to increase the risk of head and neck cancers [15], and Jawarowska et al. reported that the CYP1B1*2 355T allele increases an individual's susceptibility to laryngeal cancer [30]. Additionally, a study on squamous cell carcinoma of the respiratory tract found a high frequency of the CYP1B1*2 355T allele in patients with lung cancer, suggesting that the presence of this allele increased the risk for developing lung cancer [31]. Finally, the CYP1B1*2 355T allele was also reported to be associated with an increased susceptibility to other cancers, such as breast and endometrial cancer [32]. In our study, we found that among individuals with a history of similar environmental exposures, those individuals carrying the CYP1B1*2 355T allele (genotype GT+TT) showed a significantly increased risk for laryngeal cancer compared to individuals with the wild type gene (GG) (Table 5). Thus, data from our study and other studies have consistently shown that the CYP1B1*2 G355T allele is linked to cancer susceptibility. Mutation allele (CYP1B1*2 355T) may also increase the risk for laryngeal cancer, possibly due to the increased catalytic activity for 17β-oestradiol 4-hydroxylation contributed by that mutation [12]. However, the mechanism underlying the effect of this allele on promoting cancer risk remains unclear and requires further investigation.

CYP1B1*3 C4326G locates at the third exon of the gene and causes a Leu432Val amino acid change in the encoded enzyme. While the CYP1B1*3 C4326G mutation has been reported to increase an individual's susceptibility to head and neck cancers [8], [33], Tai et al. reported that this mutation was not associated with susceptibility to either hypopharynx squamous cell carcinoma or laryngeal cancer [28]. However, another study found a lower frequency of mutation allele CYP1B1 4326G in patients with colon cancer, indicating that this allele may reduce the risk of colon cancer and even exert a protective effect [34]. It is entirely possible that the discrepancies found among these results concerning the effect of mutation allele CYP1B1 4326G on cancer risk might derive from the different ethnicities, subject numbers, and additional SNPs included in the studies.

In our study, mutation allele CYP1B1 4326G was associated with a decreased risk for laryngeal cancer (Table 3). One possible mechanism underlying this protective effect might be that the altered enzyme conformation produced by the amino acid substitution may lead to a decreased or diminished enzymatic capacity for activating carcinogens.

CYP2E1*5 G-1293C, also known as the Rsa I/Pst I restriction fragment length polymorphism (RFLP), locates at the 5′ UTR (untranslated region) of the CYP2E1 gene. The nucleotide change G-1293C creates a recognition site for the restriction enzyme Pst I, and disrupts an Rsa I recognition site, thus altering gene expression [21], [35]. A meta-analysis including 12562 cases was performed to examine the association between the CYP2E1 Rsa I/Pst I RFLP and susceptibility to head and neck cancers [36]. The study results showed that the OR for individuals carrying one mutation allele (CYP2E1*5 -1293C) and having a head and neck cancer was 1.11 compared to individuals with the homozygous wild-type CYP2E1*5 -1293G allele. This result indicates that the CYP2E1*5 -1293C allele is associated with an increased risk for head and neck cancer. Another meta-analysis of case-control studies on the association between CYP2E1 polymorphisms and head and neck cancer risk showed that individuals, especially Asians, homozygous for CYP2E1*5 -1293C had an increased risk for developing head and neck cancer [37]. Our case-control study showed that the CYP2E1*5 -1293C allele is associated with an increased risk for laryngeal cancer, and is therefore a genetic factor for laryngeal cancer susceptibility (Table 3).

Our haplotype analyses showed that G_355_C_4326_G_−1293_ was the most common haplotype found in both the patient and control groups (Table 4), and also confirmed that both mutation alleles, CYP1B1*2 355T and CYP2E1*5 -1293C, are high risk genetic factors for developing laryngeal cancer, while the mutation allele CYP1B1*3 4326G is a protective genetic factor. These findings suggest that the wild type allele CYP1B1*3 4326C might be a high risk genetic factor for laryngeal cancer. The haplotype T_355_C_4326_C_−1293_, which includes all three high risk alleles, was associated with a significantly higher risk for laryngeal cancer compared to other haplotypes and other single-locus genotypes, suggesting the role a gene-gene interaction in development of laryngeal cancer. Haplotype G_355_G_4326_G_−1293_ was associated with a lower laryngeal cancer risk than haplotype G_355_C_4326_G_−1293_, which is consistent with the protective effect of the CYP1B1*3 4326G allele, and also suggested an additive genetic effect among these alleles. Our analyses of these haplotypes and genotypes showed that the laryngeal cancer risk increased along with increasing numbers of high risk alleles, and decreased when more protective alleles were present. These analyses not only confirmed the risk effect of each individual allele as seen by single allele analyses, but also suggested an additive joint effect of all three alleles.

Epidemiological studies have shown that environmental factors, especially smoking and alcohol consumption, play important roles in cancer development. Pro-carcinogens such as PAH, HAA, aromatic amines, N-nitrosamine, and nitrated polycyclic aromatic hydrocarbons derived from tobacco and acetaldehyde produced by alcohol metabolism can be activated and be metabolized to form carcinogens by various cytochrome P450 enzymes [38], [39]. Our study results showed that smoking and alcohol consumption, especially heavy smoking and high alcohol consumption, are associated with a significantly increased risk for developing laryngeal cancer (Table 5). Analyses for the joint effect of the three SNPs plus smoking/drinking on the risk for laryngeal cancer revealed that smokers with two of the aforementioned high risk alleles (mutation allele CYP1B1*2 355T and wild type allele CYP1B1*3 4326C) were at a significantly increased risk for developing laryngeal cancer (Table 5). Additionally, this risk was much greater than the risk conferred by either factor alone, suggesting that when combined with tobacco exposure, the two alleles had a synergistic effect for increasing the risk for laryngeal cancer. CYP1B1 is an inducible metabolic enzyme responsible for activation of multiple pro-carcinogens, and CYP1B1 translation is regulated by aryl hydrocarbon receptor (AhR), AhR nuclear translocator (ARNT), and Sp1 transcription factor [32]. Thus, the higher risk for laryngeal cancer found in smokers or drinkers carrying alleles CYP1B1*2 355T and CYP1B1*3 4326C might result from the fact that exposure to tobacco or alcohol not only leads to high cellular levels of pro-carcinogens derived from the tobacco and alcohol metabolism, but also results from increased expression and activity of P-450 metabolic enzymes that are modulated through various positive feedback mechanisms. Chemicals such as ethanol, aromatic agents, and nitrosamine derived from tobacco and alcohol metabolism are also substrates and inducers for CYP2E1 [40]; however, only a limited number of studies have been conducted examining the cross-interactions between genetic factors and smoking or drinking. Our data showed that the joint effects of CYP2E1*5 G-1293C and smoking or drinking on laryngeal cancer risk are not significantly different from the effects of individual genetic or environmental factors (Table 5). Additionally, our results showed no collaborative effect between CYP2E1*5 G-1293C and smoking/drinking, which is consistent with the results of a meta-analysis reported by Tang et al. [37].

It is also noticed that the numbers of study subjects with some genotypes such as CYP1B1*2 355TT and CYP1B1*3 4326GG are small although total case volume is relative large in our study. This limitation caused by these rare variant alleles inevitably restricted statistical power to detect low ORs in current study. Further studies with larger case volume are needed to overcome this limitation.

In summary, our case-control study showed that genetic polymorphisms of CYP1B1 and CYP2E1 are closely associated with an individual's susceptibility to developing laryngeal cancer. Analyses for the effect of each individual allele and the combined effect of multiple alleles demonstrated that mutation alleles CYP1B1*2 G355T and CYP2E1*5 G-1293C are associated with an increased risk for developing laryngeal cancer, and mutation allele CYP1B1*3 4326G exerts a protective effect against laryngeal cancer. An additive joint effect was conferred by having multiple high risk alleles, and presented as a higher laryngeal cancer risk with the more risky alleles. Our results show that both smoking and drinking play important roles in laryngeal cancer development, and are associated with higher risks with increasing levels of exposure. Finally, the combination of genetic and environmental factors (smoking and drinking) produced a synergistic effect rather than a simple additive effect on susceptibility to laryngeal cancer.
